# Supplementary material for: The efficacy of Personalized Normative Feedback interventions across addictions: A systematic review and meta-analysis
Source: PLoS One. 2021 Apr 1;16(4):e0248262. doi: 10.1371/journal.pone.0248262 (PMC8016245; doi:10.1371/journal.pone.0248262)
Supplement: S1 Appendix — (DOCX) [file pone.0248262.s002.docx]

### S1 Appendix: Search terms for literature search

A1: Search terms for PsycINFO

| ID | Search |
| --- | --- |
| #1 | Norm.mp |
| #2 | Norms.mp |
| #3 | Normative.mp |
| #4 | Social norms/ |
| #5 | Feedback.mp or FEEDBACK/ |
| #6 | “self other”.mp |
| #7 | Bibliotherapy.mp or Exp BIBLIOTHERAPY |
| #8 | #4 or #5 or #6 or #7 |
| #9 | Gambling/ or Pathological Gambling/ or Gambl*.mp |
| #10 | ALCOHOL ABUSE/ or ALCOHOL DRINKING PATTERNS/ or alcohol*.mp or ALCOHOL DRINKING ATTITUDES/ |
| #11 | BINGE DRINKING/ or “binge drink”.mp |
| #12 | Cannabis.mp or exp CANNABIS/ |
| #13 | Marijuana.mp |
| #14 | Marihuana.mp |
| #15 | Exp OPIATES/ or opiate*.mp |
| #16 | Opiod.mp |
| #17 | Exp COCAINE or cocaine*.mp |
| #18 | Exp SEDATIVES or sedative*.mp |
| #19 | Exp Benzodiazepines or benzodiazepine*.mp |
| #20 | Exp Amphetamines or amphetamine*.mp |
| #21 | Designer drugs/ |
| #22 | Exp Hallucinogenic drugs/ or hallucinogen*.mp |
| #23 | KETAMINE/ or ketamine*.mp |
| #24  #25  #26 | exp "Tobacco Use Disorder"/  exp Smoking Cessation/ or smoking.mp.  9 or 10 or 11 or 12 or 13 or 14 or 15 or 16 or 17 or 18 or 19 or 20 or 21 or 22 or 23 or 24 or 25 |
| #27 | Intervention.mp |
| #28 | Prevention.mp |
| #29 | “Comparison group”.mp |
| #30 | Trial*.mp |
| #31 | Random*.mp |
| #32 | Control*.mp |
| #33 | Treatment*.mp |
| #34 | 27 or 28 or 29 or 30 or 31 or 32 or 33 |
| #35 | 27 or 28 or 29 or 30 or 31 or 32 or 33 |
| #36  #37 | limit 35 to (human and english language and yr="2000 - 2019")  limit 36 to peer reviewed journal |

A2: Search terms for Medline

| ID | Search |
| --- | --- |
| #1 | SOCIAL NORMS/ or norms.mp |
| #2 | Norms.mp |
| #3 | Normative.mp |
| #4 | Feedback.mp |
| #5 | FEEDBACK, PSYCHOLOGICAL/ |
| #6 | “self other”.mp |
| #7 | Biobliotheraphy.mp |
| #8 | #1 or #2 or #3 or #4 or #5 or #6 or #7 |
| #9 | Gambling/ or gambl*.mp |
| #10 | Alcohol-related disorders/ or alcohol-induced disorders/ or alcohol intoxication/ or alcoholism/ or binge drinking/ |
| #11 | Drinking behavior/ or alcohol abstinence/ or alcohol drinking/ or binge drinking/ or alcohol drinking in college/ or underage drinking/ |
| #12 | Substance-related disorders/ or amphetamine-related disorders/ or cocaine-related disorders/ or drug overdose/ or heroin dependence/ or inhalant abuse/ or marijuana abuse/ or opioid-related disorders/ or morphine dependence/ or opium dependence/ or phencyclidine abuse or psychoses, substance-induced/ or substance-abuse, intravenous/ or substance-abuse, oral/ |
| #13 | Drug misuse/ or prescription drug misuse/ or prescription drug overuse/ |
| #14 | “marijuana use”/ or marijuana smoking/ |
| #15 | Street drugs/ or crack cocaine/ |
| #16 | Designer Drugs/ |
| #17 | Benzodiazepine*.mp |
| #18 | Ketamine.mp |
| #19 | Cocaine.mp |
| #20 | Sedative*.mp |
| #21 | Narcotic*.mp |
| #22  #23  #24 | exp Tobacco Smoking/ or exp "Tobacco Use Disorder"/ or exp "Tobacco Use Cessation"/ or tobacco.mp.  exp Smoking Cessation/ or exp Smoking Reduction/ or smoking.mp. or exp Smoking/  9 or 10 or 11 or 12 or 13 or 14 or 15 or 16 or 17 or 18 or 19 or 20 or 21 or 22 or 23 |
| #25 | Intervention.mp |
| #26 | Prevention.mp |
| #27  #28 | Treatment*.mp  “Comparison group”.mp |
| #29 | Trial*.mp |
| #30 | Random*.mp |
| #31 | Control*.mp |
| #32 | 25 or 26 or 27 or 28 or 29 or 30 or 31 |
| #33 | 8 and 24 and 32 |
| #34 | limit 33 to (english language and humans and yr="2000 -2019") |
| #35 | limit 34 to randomized controlled trial |

A3: Search terms for Embase

| ID | Search |
| --- | --- |
| #1 | Social norms/ |
| #2 | Norm.mp |
| #3 | Norms.mp |
| #4 | Normative.mp |
| #5 | Feedback.mp |
| #6 | “Self other”.mp |
| #7 | Bibliotherapy.mp |
| #8 | #1 or #2 or #3 or #4 or #5 or #6 or #7 |
| #9 | Gambling/ or gambl*.mp or pathological gambling/ |
| #10 | Alcohol*.mp or alcohol consumption/ |
| #11 | Alcohol abuse/ or binge drinking/ or college drinking/ or underage drinking/ |
| #12 | Drinking behavior/pc, th [Prevention, Therapy] |
| #13 | Drug abuse/ or alcohol abuse/ or amphetamine abuse/ or analgesic agent abuse/ or drug abuse pattern/ or drug misuse/ or illicit drug inhalation/ or inhalant abuse/ or intravenous drug abuse/ or multiple drug abuse/ or phencyclidine abuse/ or prescription drug diversion/ |
| #14 | Cannabis addition/pc, th [Prevention, Rehabilitation, Therapy] |
| #15 | Drug dependence/ or amphetamine dependence/ or benzodiazepine dependence/ or cannabis addiction/ or cocaine dependence/ or congenital drug dependence/ or drug abuse pattern/ or drug craving/ or drug misuse/ or drug seeking behavior/ or glue sniffing/ or methamphetamine dependence/ or multiple drug abuse/ or narcotic dependence/ or phencyclidine dependence/ |
| #16 | Drug misuse/pc, th [Prevention, Rehabilitation, Therapy] |
| #17 | Narcotic dependence/ or narcotic*.mp |
| #18 | Sedative*.mp |
| #19 | Opiate*.mp |
| #20 | Benzodiazepine*.mp |
| #21  #22  #23 | Ketamine.mp  exp tobacco dependence/ or exp "tobacco use"/ or tobacco.mp. or exp tobacco smoke/  exp smoking cessation/ or smoking.mp. or exp smoking/ or exp smoking cessation program/ or exp smoking reduction/ |
| #24 | 9 or 10 or 11 or 12 or 13 or 14 or 15 or 16 or 17 or 18 or 19 or 20 or 21 or 22 or 23 |
| #25 | Intervention.mp |
| #26 | Prevention.mp |
| #27 | “Comparison group”.mp |
| #28 | Trial*.mp |
| #29 | Random*.mp |
| #30 | Control*.mp |
| #31 | Treatment*.mp |
| #32 | 25 or 26 or 27 or 28 or 29 or 30 or 31 |
| #33 | 8 and 24 and 32 |
| #34 | Limit #34 to (humans and English language and last 19 years) |
| #35 | Limit #35 to Journal |
| #36 | Limit #36 to Randomized Controlled Trial |

A4: Search terms for the Cochrane Library

ID Search

#1 "norm"

#2 "norms"

#3 "normative"

#4 MeSH descriptor: [Social Norms] this term only

#5 feedback

#6 MeSH descriptor: [Feedback, Psychological] explode all trees

#7 "self other"

#8 bibliotherapy

#9 #1 or #2 or #3 or #4 or #5 or #6 or #7 or #8

#10 gambl*

#11 MeSH descriptor: [Gambling] this term only

#12 MeSH descriptor: [Drinking Behavior] explode all trees

#13 MeSH descriptor: [Substance-Related Disorders] 2 tree(s) exploded

#14 MeSH descriptor: [Tobacco Use Disorder] explode all trees

#15 MeSH descriptor: [Marijuana Use] explode all trees

#16 ketamine

#17 benzodiazapine*

#18 cocaine

#19 sedative*

#20 narcotic*

#21 MeSH descriptor: [Smoking Cessation] explode all trees

#22 MeSH descriptor: [Smoking Reduction] explode all trees

#23 MeSH descriptor: [Tobacco Use Cessation] explode all trees

#24 smoking

#25 #10 OR #11 OR #12 OR #13 OR #14 OR #15 OR #16 OR #17 OR #18 OR #19 OR #20 OR #21 OR #22 OR #23 OR #24

#26 intervention*

#27 prevention*

#28 treatment*

#29 trial*

#30 random*

#31 "comparison group"

#32 control*

#33 #26 OR #27 OR #28 OR #29 OR #30 OR #31 OR #32

#34 #9 AND #25 AND #33

with Publication Year from 2000 to 2019, in Trials
