# Supplementary material for: The efficacy of Personalized Normative Feedback interventions across addictions: A systematic review and meta-analysis
Source: PLoS One. 2021 Apr 1;16(4):e0248262. doi: 10.1371/journal.pone.0248262 (PMC8016245; doi:10.1371/journal.pone.0248262)
Supplement: S2 Appendix — (DOCX) [file pone.0248262.s003.docx]

### S2 Appendix: Decision rules for the review and meta-analysis

1. Studies with more than one available frequency and/or severity outcome measure:

- All data for those outcomes will be extracted and included in the ‘characteristics of included studies’ tables
- Only the highest ranked frequency and severity outcomes will be included in the meta-analysis, per time point (see prioritization details below)
- Frequency outcomes:
  - May be presented as ‘days’ ‘occasions’ or ‘episodes’
  - If more than one frequency outcome is available in a paper, we will prioritize as follows: 1) days 2) occasions 3) episodes.
- Severity outcomes:
  - A range of tools are available with the potential to create high heterogeneity in our meta-analyses. To maximize uniformity/comparability between papers, we will prioritize as follows:
    - Most commonly used tools (AUDIT and Rutgers Alcohol Problem Index) over those rarely used;
    - Complete tools rather than selected subscales (AUDIT10 preferred over AUDIT-C);
    - Standardized tools over un-standardized;
    - Multi-dimensional tools over unidimensional or those with fewer dimensions.

1. Data reporting rules:

- Un-standardized data will be reported in the ‘characteristics of included studies’ tables
- Standardized data will be reported in the meta-analyses (standardized mean differences)

1. A minimum of two studies per meta-analysis is required
2. Preferences if multiple statistics are reported in a single study:

- Mean and SD are preferred over all other statistics (e.g. effect size)
- Final value mean scores are preferred over mean change from baseline scores
- If ITT and completer data are both available, we will use ITT
- If results from multiple intervention arms are presented overall and separately, we will use the latter
- In studies with multiple PNF arms, data from all arms will be included. A single mean and SD representing all intervention arms will be calculated using the formulae in the Cochrane handbook; numbers of participants in each arm will be summed.
- If no unadjusted analyses are available, we will use the least adjusted model (with least covariates)
- If results are available for males and females separately and overall we will use the overall results.
- If results are presented for females and males separately, but not overall, we will use both values
- If an article presents data for more than one participant group (e.g. moderate drinker, heavy drinker) and does not present overall participant data (moderate and heavy drinkers combined), we will use the more extreme participant group (e.g. heavy drinker).
- If an article presents two or more values within a single follow up period (as defined in our review), we will use data from the longest follow up.

1. If multiple papers are available for the same data set we will:

- Use the article with the highest ranking outcomes
- Use the article with the longest follow-up period
- Use the article with unadjusted/least adjusted results
- Use the article reporting on the most robust measurement tool

1. Articles missing key information:

- If the analytic sample size is not reported we will use the baseline n
- If sample size by group is not reported we will exclude the study from the meta-analyses but report results in the ‘characteristics of included studies’ tables
- If mean and SD are not reported, we will calculate them as per the conversion formulae available in the Cochrane handbook^[[1]](#footnote-1)^
- We will extract data from graphs where resolution permits, in the absence of data tables.
- We will convert medians to means as per published guidance^[[2]](#footnote-2)^ .
- Data we cannot convert to means or SDs for our meta-analyses will be reported in the ‘characteristics of included studies’ tables.

1. Higgins JPT & Green,S (eds) (2011). Data extraction for continuous outcomes *Cochrane Handbook for Systematic Reviews of Interventions Version 5.1.0*: The Cochrane Collaboration, 2011. [↑](#footnote-ref-1)
2. Hozo, S. P., Djulbegovic, B., & Hozo, I. (2005). Estimating the mean and variance from the median, range, and the size of a sample. *BMC Medical Research Methodology, 5*(1), 13. doi:10.1186/1471-2288-5-13 [↑](#footnote-ref-2)
