# Supplementary material for: The efficacy of Personalized Normative Feedback interventions across addictions: A systematic review and meta-analysis
Source: PLoS One. 2021 Apr 1;16(4):e0248262. doi: 10.1371/journal.pone.0248262 (PMC8016245; doi:10.1371/journal.pone.0248262)
Supplement: S3 Appendix — (DOCX) [file pone.0248262.s004.docx]

### S3 Appendix: Description of the interventions

#### PNF content

Intervention formats for the PNF component were broadly similar across studies. All studies presented feedback to participants in the form of graphs and text: (1) their self-reported behavior (e.g., number of alcoholic drinks consumed per week); (2) the participant’s perception of their peers’ behavior (e.g., average number of alcoholic drinks perceived to be consumed by their peers); and (3) the actual descriptive norm (e.g., the average number of alcoholic drinks consumed by the participant’s peer group). Ten studies (30%) also presented participants with their percentile rank for the behavior (i.e., where they were positioned in relation to their peers).

The dimensions of behavior for which descriptive normative feedback were provided tended to focus on frequency and quantity of consumption. In mixed PNF studies, normative feedback was often also given about the secondary effects of a behavior (e.g., alcohol calories consumed, money spent, and average blood alcohol levels) (Collins et al. 2014). Misperceptions of descriptive norms were explicitly highlighted in two trials (Neighbors et al. 2016; Lewis et al. 2017) which included the message: “most students don’t drink as much as you think they do”.

Only three of the 34 included trials (9%) included injunctive norms in their feedback. One study measured participants’ beliefs about whether close friends would approve/disapprove/not care about various dimensions of their marijuana use (Elliott et al. 2014). Another study measured participant’s perceptions of university peers’ approval of alcohol use, where phrasing of the feedback explicitly highlighted discrepancies in participants’ perceptions of injunctive norms (Ridout & Campbell 2014). The third study varied PNF content across eight intervention arms by type of message framing, including whether a behavior was positively or negatively viewed by others (Neighbors et al. 2018).

Several studies varied the specificity of the reference groups that participants were compared against on the focus behavior, for example, by examining whether gender-specific or gender-neutral feedback made a difference to intervention efficacy (Lewis & Neighbors 2007; Lewis et al. 2007). LaBrie et al. (2013) varied their reference groups according to gender, membership of particular college groups, and ethnicity. Two studies assessed whether single or repeated PNF gave different results (Neighbors et al. 2010; Andersson et al. 2015), whilst others used booster sessions, providing participants with feedback comparing their drinking levels at the one-month interim follow up period with baseline drinking (Kypri et al. 2009, Ridout & Campbell 2014). Palfai et al. 2014 compared the efficacy of onsite and offsite-delivered PNF. Finally Elliott et al. (2014) tested whether initial assessments made a difference to cannabis use, where one PNF group did, and the other did not have marijuana use measured at the assessment stage.

#### Additional intervention content (mixed PNF studies only)

We grouped additional intervention content into five categories, summarized in the text and Table 1 below.

1. Presentation of negative consequences

Presentation of negative consequence was the most common addition to PNF. Seventeen studies (71%) provided personalized negative health consequences associated with the addictive behavior, such as a personal risk level for liver disease. Thirteen studies (54%) included estimates of participants’ blood alcohol content and associated health problems. Nine studies (38%) presented negative social and behavioral consequences or risks associated with the behavior (e.g., of traffic accident). Eight studies (33%) estimated the calories consumed by the participant through alcohol, and weight gain implications. Three studies (13%) provided participants with feedback on other harmful self-reported health behaviors (e.g., tobacco use), and a further three provided participants with diagnostic labels associated with their health behavior (e.g., problem gambling).

1. Financial and/or time costs associated with the behavior

Linked to the negative consequences described above, 14 studies (58%) provided estimates of participants’ expenditure associated with the health behavior (e.g., monthly or yearly costs). Two studies (8%) gave feedback on the amount of time participants were spending engaged with the behavior versus other activities.

1. Tips and tools for cutting down

Twelve studies (50%) provided tips and tools for cutting down on the behavior. Two of these studies included goal setting tools, and five provided suggestions of alternative ways to spend money.

1. Information provision

Five studies (21%) presented official guidelines (e.g., for drinking), standard drink sizes, or criteria for dependence. A further five studies provided general information about the problem behavior (e.g., on alcohol and health). Seven studies (29%) provided contact information for referral and treatment services, and other resources to address problem use.

1. Exploration of participant’s current feelings and opinions about their behavior

Seven studies (29%) provided feedback on participants’ self-reported concerns about their behavior, explored pros and cons, or used cost-benefit scales. Two studies (8%) assessed participants’ readiness to change their behavior, and a further two gave feedback on self-reported gambling myths, cognitive distortions, or alcohol expectancies. One recent study included a 15-20 minute expressive writing task following PNF, asking participants to write down how they felt about their drinking in light of their feedback, and whether they planned to reduce their alcohol intake (Young & Neighbors 2019).

#### References

Andersson C. Comparison of WEB and Interactive Voice Response (IVR) methods for delivering brief alcohol interventions to hazardous-drinking university students: A randomized controlled trial. European Addiction Research. 2015;21(5):240-52.

Collins SE, Kirouac M, Lewis MA, Witkiewitz K, Carey KB. Randomized controlled trial of web-based decisional balance feedback and personalized normative feedback for college drinkers. Journal of Studies on Alcohol and Drugs. 2014;75(6):982-92.

Elliott JC, Carey KB, Vanable PA. A preliminary evaluation of a web-based intervention for college marijuana use. Psychology of Addictive Behaviors. 2014;28(1):288-93.

Kypri K, Hallett J, Howat P, McManus A, Maycock B, Bowe S, et al. Randomized controlled trial of proactive web-based alcohol screening and brief intervention for university students. Archives of Internal Medicine. 2009;169(16):1508-14.

LaBrie JW, Lewis MA, Atkins DC, Neighbors C, Zheng C, Kenney SR, et al. RCT of web-based personalized normative feedback for college drinking prevention: Are typical student norms good enough? Journal of Consulting and Clinical Psychology. 2013;81(6):1074-86.

Lewis MA, Litt DM, Tomkins M, Neighbors C. Prototype willingness model drinking cognitions mediate personalized normative feedback efficacy. Prevention Science. 2017;18(4):373-81.

Lewis M, Neighbors C. Optimizing personalized normative feedback: the use of gender-specific referents. Journal of studies on alcohol and drugs. 2007; 68(2):[228-37 pp.].

Lewis MA, Neighbors C, Oster-Aaland L, Kirkeby BS, Larimer ME. Indicated prevention for incoming freshmen: Personalized normative feedback and high-risk drinking. Addictive Behaviors. 2007;32(11):2495-508.

Neighbors C, DiBello AM, Young CM, Steers MLN, Rinker DV, Rodriguez LM, et al. Personalized normative feedback for heavy drinking: An application of deviance regulation theory. Behaviour Research and Therapy. 2018.

Neighbors C, Lewis MA, Atkins DC, Jensen MM, Walter T, Fossos N, et al. Efficacy of web-based personalized normative feedback: A two-year randomized controlled trial. Journal of Consulting and Clinical Psychology. 2010;78(6):898-911.

Neighbors C, Lewis MA, LaBrie J, DiBello AM, Young CM, Rinker DV, et al. A multisite randomized trial of normative feedback for heavy drinking: Social comparison versus social comparison plus correction of normative misperceptions. Journal of Consulting & Clinical Psychology. 2016;84(3):238-47.

Palfai TP, Saitz R, Winter M, Brown TA, Kypri K, Goodness TM, et al. Web-based screening and brief intervention for student marijuana use in a university health center: pilot study to examine the implementation of eCHECKUP TO GO in different contexts. Addictive Behaviors. 2014;39(9):1346-52.

Ridout B, Campbell A. Using Facebook to deliver a social norm intervention to reduce problem drinking at university. Drug and alcohol review. 2014; 33(6):[667-73 pp.].

Young CM, Neighbors C. Incorporating Writing into a Personalized Normative Feedback Intervention to Reduce Problem Drinking Among College Students. Alcohol Clin Exp Res. 2019;43(5):916-26.

### S3C Table 1: Additional intervention components used in mixed PNF studies, and number of studies incorporating each component

|  | **Intervention component** | **No of studies** |
| --- | --- | --- |
| ***Negative consequences of health behavior*** | |  |
| 1 | Labelling participant as problem user e.g. provide their gambling category | 3 |
| 2 | Estimates of blood alcohol content | 13 |
| 3 | Presentation of negative health consequences / personal risk level e.g. liver disease | 17 |
| 4 | Presentation of negative social and behavioral consequences or risks (e.g. of traffic accident) | 9 |
| 5 | Calories consumed as alcohol, and weight gain implications | 8 |
| 6 | Feedback on other potentially harmful health behaviors e.g. cigarettes | 3 |
| ***Financial and time implications of their behavior*** | |  |
| 7 | Expenditure (e.g. monthly on alcohol) | 14 |
| 8 | Feedback on time spent engaged in behavior | 2 |
| ***Tips and tools for cutting down*** | | |
| 9 | Goal setting (e.g. to cut down) | 2 |
| 10 | Other tips and tools on how to cut down or reduce risk | 12 |
| 11 | Suggestions of alternative ways to spend money | 5 |
| ***Information provision*** | | |
| 12 | Presentation of official guidelines (e.g. for drinking), standard drink sizes, criteria for dependence | 5 |
| 13 | General information about alcohol and health | 5 |
| 14 | Contact information for treatment/referral services, and other resources | 7 |
| ***Exploration of participant’s current feelings and wider views about their behavior*** | |  |
| 15 | Review of participant’s self-reported concerns, pros and cons, cost-benefit scales | 6 |
| 16 | Assessment of participant’s readiness to change | 2 |
| 17 | Review of self-reported gambling myths, cognitive distortions, or alcohol expectancies | 2 |
| 18 | Expressive writing task: Participants asked to write for 15-20 minutes about their reactions to PNF (e.g., how they felt about their drinking after reviewing the feedback, if they had plans to alter their drinking habits) | 1 |
