# Supplementary material for: The efficacy of Personalized Normative Feedback interventions across addictions: A systematic review and meta-analysis
Source: PLoS One. 2021 Apr 1;16(4):e0248262. doi: 10.1371/journal.pone.0248262 (PMC8016245; doi:10.1371/journal.pone.0248262)

### S4 Appendix: Pure PNF: subgroup and sensitivity analyses

#### Frequency: Subgroup analyses

#### Addiction type: Alcohol

Subgroup analysis of the five alcohol studies indicated significantly lower alcohol frequency at 0-3 months post-baseline in the pure PNF group compared to the control groups. While the effect size was small, the results indicated a beneficial effect of pure PNF (SMD -0.22, 95%CI -0.40 to -0.04). Heterogeneity across studies was substantial (I^2^=69%; Chi² = 12.81, p = 0.01). The same analyses for the 4-11 (k=5; SMD -0.14, 95%CI -0.30 to 0.02) and 12-23 (k=2; SMD -0.02, 95%CI -0.15 to 0.11) month follow up periods returned non-significant results. While heterogeneity was substantial in the 4-11 month group (I^2^=63%; Chi² = 10.92, p = 0.03), it was minimal in the 12-23 month group (I^2^=0%; Chi² = 0.14, p = 0.71).

There were insufficient studies to conduct further subgroup analyses, relating to addiction or sample type.

#### Frequency: Sensitivity analyses

#### Overall risk of bias

A sensitivity analysis, in which the study with ‘some concerns’ about risk of bias was removed from the 0-3 month follow up analysis, was conducted. The results remained non-significant (SMD=-0.13, 95%CI -0.23 to 0.03), however, heterogeneity was substantially reduced (I^2^=9%; Chi² = 3.28, p = 0.35). There were insufficient studies available to conduct further sensitivity analyses on other follow up periods or studies with medians converted to means.

#### Symptom severity: Subgroup analyses

#### Addiction type: Alcohol

Subgroup analysis of ten alcohol studies for the 0-3 month follow up period resulted in a small but significant difference in symptom severity, favoring the pure PNF group (SMD -0.07, 95% CI -0.14 to -0.004). Heterogeneity was minimal (I² = 0%; Chi² = 4.58, p = 0.87). Subgroup analyses of alcohol studies for further follow up periods showed no significant difference in symptom severity between PNF and control groups. These analyses consisted of six studies for the 4-11 month follow up period (SMD 0.04, 95% CI -0.07 to 0.14), and two studies for the 12-23 month follow up period (SMD 0.13, 95% CI -0.01 to 0.26), both with minimal heterogeneity (4-11 months: I² = 35%; Chi² = 7.74, p = 0.17; 12-23 months: I² = 0%; Chi² = 0.07, p = 0.79).

#### Sample type: College/University settings

When only studies from college/university settings were included (10/11 possible studies), the SMD indicated a small but significant difference in short-term symptom severity, favoring the pure PNF group (SMD =-0.10, 95%CI -0.17 to -0.02), where heterogeneity was minimal (I² = 0%; Chi² = 2.32, p = 0.99).

There were insufficient studies to conduct further subgroup analyses, relating to addiction or sample type.

#### Symptom severity: Sensitivity analyses

#### Overall risk of bias

In sensitivity analyses in which we excluded the three studies deemed to have ‘some concerns’ over risk of bias in the 0-3 month follow up group, the SMD remained significant, strengthening slightly, again suggesting a small difference in symptom severity, favoring the pure PNF group (SMD=-0.10, 95%CI -0.17 to -0.02). Heterogeneity was unchanged (I² = 0%; Chi² = 4.62, p = 0.92). There were insufficient studies available to conduct further sensitivity analyses by follow up period.

### Forest plots for pure PNF studies

D1: Pure PNF vs passive control: 0-3 month follow up

D1.1 Frequency of behavior


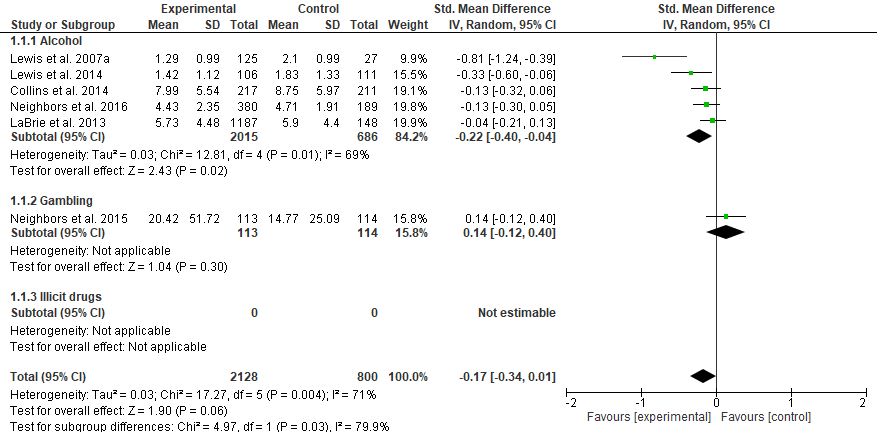


D1: Pure PNF vs passive control: 0-3 month follow up

D1.2 Symptom severity


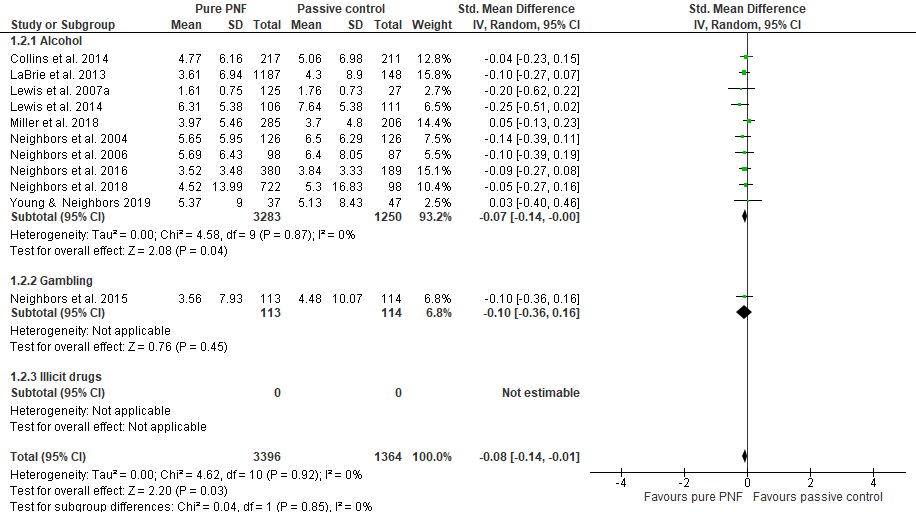


D2: Pure PNF vs passive control: 4-11 month follow up

D2.1 Frequency of behavior


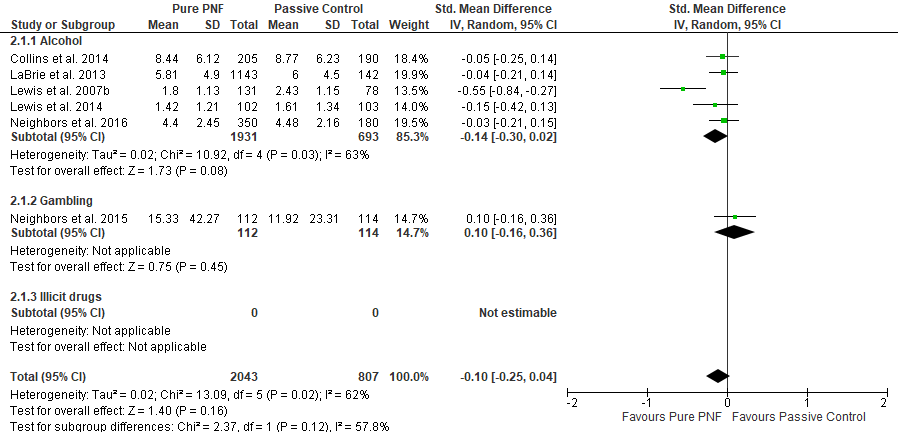


D2: Pure PNF vs passive control: 4-11 month follow up

D2.2 Symptom severity


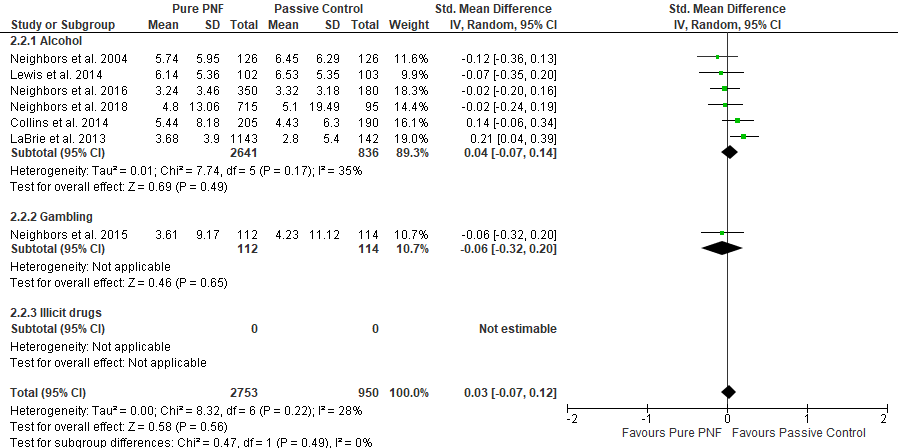


D3: Pure PNF vs passive control: 12-23 month follow up

D3.1 Frequency of behavior


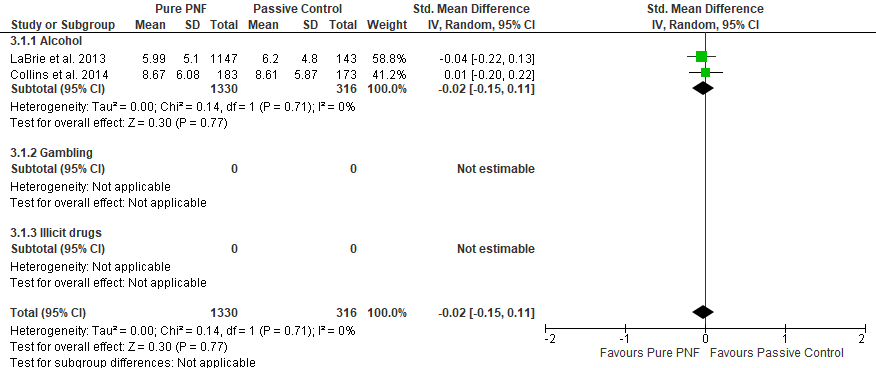


D3: Pure PNF vs passive control: 12-23 month follow up

D3.2 Symptom severity


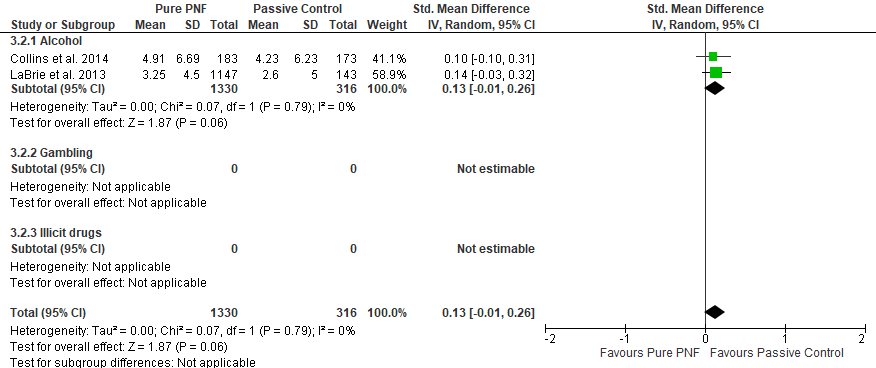

Supplement: S4 Appendix — (DOCX) [file pone.0248262.s005.docx]
