# Supplementary material for: The efficacy of Personalized Normative Feedback interventions across addictions: A systematic review and meta-analysis
Source: PLoS One. 2021 Apr 1;16(4):e0248262. doi: 10.1371/journal.pone.0248262 (PMC8016245; doi:10.1371/journal.pone.0248262)

### S5 Appendix: Mixed PNF: subgroup and sensitivity analyses

##### Frequency: Subgroup analyses

##### Addiction type: Alcohol

At 0-3 months, meta-analysis of seven alcohol studies resulted in significantly lower frequency in the mixed PNF group relative to the control (SMD -0.12, 95% CI -0.20 to -0.03), with minimal heterogeneity (I² = 36%; Chi² = 9.38, p = 0.15) and a small effect size. At 4-11 months, the pooled results from six alcohol studies again indicated a small but significant difference in frequency of alcohol consumption amongst mixed PNF participants compared to controls (SMD -0.11, 95% CI -0.20 to -0.03, but with substantial heterogeneity (I² = 61%; Chi² = 12.66, p = 0.03). There were two studies for the 12-23 month follow up period for which there was no significant effect of mixed PNF on frequency (SMD -0.01, 95% CI -0.14 to 0.12), with minimal heterogeneity (I² = 0%; Chi² = 0.09, p = 0.77).

#### Addiction type: Gambling

The two gambling studies available for meta-analysis at 0-3 months resulted in no intervention effect on frequency (SMD 0.00, 95% CI-0.22 to 0.23), with low heterogeneity (I² = 0%; Chi² = 0.26, p = 0.61). There were insufficient studies to conduct further gambling subgroup analyses on other follow up periods.

#### Addiction type: Illicit drug use

The two illicit drug (cannabis) studies for the 0-3 month follow up period indicated no mixed PNF effect on reducing frequency (SMD -0.02, 95% CI -0.20 to 0.15), with low heterogeneity (I² = 0%; Chi² = 0.22, p = 0.64). There were insufficient studies to conduct further illicit drug use subgroup analyses on other follow up periods.

#### Setting: College/University

When we restricted our analyses to the ten studies conducted in university settings, with 0-3 months follow up periods, the result remained: there was a small but significant difference in frequency that favored the mixed PNF group compared to the control (SMD -0.10 95%CI -0.16 to -0.03), with minimal heterogeneity (I² = 16%; Chi² = 10.66, p = 0.30;). At 4-11 months, when we restricted our analysis to the six studies conducted in university settings, the result indicated significantly lower frequency amongst PNF participants compared to controls (SMD -0.12, 95%CI -0.21 to -0.04), with moderate heterogeneity (I² = 53%; Chi² = 10.68, p = 0.06;). There were insufficient studies to conduct setting-related subgroup analyses for the 12-23 month follow up period.

#### Additional intervention components:

All studies with 0-3 month follow up periods presented negative consequences of behavior, so it was not possible to examine whether this component influenced PNF efficacy. Five studies presented financial and time costs associated with the behavior, which resulted in a non-significant difference in frequency (SMD -0.13, 95%CI -0.25 to 0.0029), with minimal heterogeneity (I² = 36%; Chi² = 6.24, p = 0.18). Tips and tools for cutting down were presented in seven studies, and returned a non-significant result for PNF (SMD -0.05, 95%CI -0.12 to 0.02) with minimal heterogeneity (I² = 0%; Chi² = 1.60, p = 0.95). Information provision formed part of five PNF interventions, and together indicated no effect of PNF (SMD -0.11, 95%CI -0.23 to 0.01) with moderate heterogeneity (I² = 48%; Chi² = 7.70, p = 0.10). Three studies incorporated exploration of participant’s current feelings and opinions about their behavior into their PNF interventions. This subgroup analysis was not significant (SMD -0.03, 95%CI -0.17 to 0.12), and there was minimal heterogeneity (I² = 0%; Chi² = 0.22, p = 0.89).

For studies with 4-11 month follow ups, all PNF interventions incorporated negative consequences and therefore we could not explore how this component influenced PNF efficacy. Five studies incorporated financial/time costs associated with the behavior, resulting in small but significant between-group differences in frequency, favoring the PNF group (SMD -0.11, 95%CI -0.20 to -0.02), with substantial heterogeneity (I² = 68%; Chi² = 12.65, p = 0.01); this effect size was similar to that derived from the main analysis. Information provision was included in five studies, and resulted in significantly different frequency scores that favored the PNF group (SMD -0.11, 95%CI -0.20 to -0.02), with substantial heterogeneity (I² = 68%; Chi² = 12.65, p = 0.01). Four studies included tips and tools for cutting down, producing a non-significant result in the subgroup analysis (SMD -0.10, 95%CI -0.26 to 0.06), with substantial heterogeneity (I² = 73%; Chi² = 10.94, p = 0.01).

Only one study included exploration of participant’s current feelings and opinions about their behavior so a subgroup analysis was not possible. No subgroup analyses exploring the influence of additional intervention components for the 12-23 month follow up period were possible due to insufficient studies.

#### Frequency: Sensitivity analyses

#### Overall risk of bias

In sensitivity analyses for the 0-3 month follow up period, with removal of the three studies with some concerns or high risk of bias (two gambling studies and one alcohol study), the result remained significant with minimal change to the SMD (-0.09, 95%CI -0.15 to -0.04), but heterogeneity decreased (I² = 0%; Chi² = 4.76, p = 0.69). In further sensitivity analyses for this follow up period, we removed the study for which we converted medians to means, and the result remained significant and marginally reduced the SMD to -0.08 (95%CI -0.15 to 0.0005), as well as slightly reducing heterogeneity (I² = 11%; Chi² = 10.16, p = 0.34). In sensitivity analyses for the 4-11 month period in which we removed the four studies (all alcohol focused) for which we converted medians to means, this substantially reduced the number per group (n=410 PNF, n=415 control). The SMD weakened to -0.06 (95%CI -0.20 to 0.08) indicating no PNF effect on frequency at this time point, and heterogeneity was lowered (I² = 0%; Chi² = 0.37, p = 0.83).

There were insufficient studies available to conduct further sensitivity analyses.

#### Mixed PNF: Symptom severity: Subgroup analyses

#### Addiction type: Alcohol

Subgroup analysis of eleven alcohol studies for the 0-3 month follow up period resulted in a small but significant difference in symptom severity (SMD -0.06, 95%CI -0.12 to -0.003), with minimal heterogeneity (I² = 0%; Chi² = 8.38, p = 0.59). For the 4-11 month follow up period, nine studies were meta-analyzed, resulting in no impact of mixed PNF on symptom severity (SMD -0.02, 95%CI -0.15 to 0.11), and with substantial heterogeneity (I² = 84%; Chi² = 51.17, p<0.0001). There were two studies available for the 12-23 month follow up period. In an identical meta-analysis as the main analysis for this time period, there was a significant between group difference that favored the control group (SMD 0.24, 95%CI 0.11 to 0.37, with minimal heterogeneity (I² = 0%; Chi² = 0.51, p = 0.47).

#### Addiction type: Gambling

The two available gambling studies for the 0-3 month follow up period indicated a small but significant between group difference in symptom severity that favored the mixed PNF group (SMD -0.26, 95%CI -0.48 to -0.03), with minimal heterogeneity (I² = 0%; Chi² = 0.73, p = 0.39). Though the effect size here was larger than observed in most of our other meta-analyses, it is worth noting that one study was judged to have some concerns, and the other a high risk of bias. There were insufficient gambling studies available to conduct subgroup analyses for longer follow up periods.

#### Addiction type: Illicit drug use

There were two illicit drug studies available for the 0-3 month follow up period. Results of the meta-analysis indicated no impact of mixed PNF on symptom severity (SMD 0.11, 95%CI -0.06 to 0.29, with minimal heterogeneity (I² = 0%; Chi² = 0.25, p = 0.62). There were insufficient illicit drug studies available to conduct subgroup analyses for longer follow up periods.

#### Setting: College/University

When we restricted our analysis of 0-3 month follow up studies to those conducted in college/university settings (14 of 15 studies), there was no significant impact of mixed PNF on symptom severity (SMD=-0.07, 95%CI -0.14 to 0.01); heterogeneity was minimal (I² = 19%; Chi² = 16.07, p = 0.25). For the 4-11 month follow up period, when we restricted our analysis to studies from university settings (nine of ten studies), the result was non-significant (SMD -0.05, 95%CI -0.16 to 0.07), and heterogeneity was substantial (I² = 77%; Chi² = 34.28, p < 0.001). There were insufficient studies available to conduct further subgroup analyses for setting by follow up period.

#### Additional interventions symptom severity:

Fourteen of the 15 studies with 0-3 month follow up periods presented negative consequences of behavior. When we restricted our analysis to these papers, the result was no longer significant (SMD=-0.07, 95%CI -0.14 to 0.004), with minimal heterogeneity (I² = 0%; Chi² = 0.25, p = 0.62). Nine studies included financial and time costs associated with the behavior in their mixed PNF interventions. Meta-analysis indicated no effect of mixed PNF (SMD -0.06, 95%CI -0.16 to 0.04), with minimal heterogeneity (I² = 16%; Chi² = 9.51, p = 0.30). Six studies included tips and tools for cutting down. The meta-analysis indicated no effect of mixed PNF (SMD -0.06, 95% -0.18 to 0.06), with moderate heterogeneity (I² = 51%; Chi² = 10.28, p = 0.07). Six studies also included information provision in their intervention. When meta-analyzed, the results suggested no impact of mixed PNF (SMD -0.02, 95%CI -0.09 to 0.06), with minimal heterogeneity (I² = 0%; Chi² = 4.86, p = 0.43). Six studies explored participants’ current feelings and opinions about their behavior. Meta-analysis results indicated no effect of mixed PNF (SMD -0.07, 95%CI -0.22 to 0.09), with moderate heterogeneity (I² = 43%; Chi² = 8.79, p = 0.12).

All studies with 4-11 month follow up periods presented negative consequences of behavior, so it was not possible to examine whether this component influenced mixed PNF efficacy. Seven studies presented financial and time costs associated with the behavior to participants. A meta-analysis of these studies suggested no impact of mixed PNF on symptom severity (SMD=0.00, 95%CI -0.16 to 0.16), with substantial heterogeneity (I² = 88%; Chi² = 50.29, p < 0.001). Five studies used tips and tools for cutting down, but the meta-analysis indicated no effect of mixed PNF (SMD=0.07, 95%CI -0.13 to 0.28), with substantial heterogeneity (I² = 92%; Chi² = 48.71, p < 0.001). Seven studies used information provision in their interventions. The meta-analysis indicated no impact of mixed PNF on symptom severity (SMD -0.02, 95%CI -0.16 to 0.13), with substantial heterogeneity (I² = 88%; Chi² = 51.11, p < 0.001). Two studies explored participant’s current feelings and opinions about their behavior, but again the meta-analysis results were non-significant for mixed PNF (SMD=0.09, 95%CI -0.10 to 0.28), with minimal heterogeneity (I² = 0%; Chi² = 0.28, p = 0.60).

#### Symptom severity: Sensitivity analyses

#### Overall risk of bias

In sensitivity analyses for the 0-3 month follow up group, in which we omitted the five studies with some concerns and high risk of bias (three alcohol studies, and both gambling studies), the SMD was -0.04 (95%CI -0.10 to 0.02). This indicates a non-significant effect of mixed PNF (as observed in the main analysis), with minimal heterogeneity (I² = 6%; Chi² = 9.55, p = 0.39). In further sensitivity analyses for this follow up period, when we removed the one study for which we converted medians to means (78), the SMD was -0.09 (95%CI -0.16 to -0.02), indicating a small but significant difference in symptom severity that favored the intervention group, where heterogeneity was minimal (I² = 5%; Chi² = 13.62, p = 0.40).

For the ten studies with 4-11 month follow ups, all were considered to have low risk of bias. In the other sensitivity analysis for this follow-up period, removal of the four studies for which we converted medians to means, changed the SMD to -0.02 (95%CI -0.19 to 0.15), with substantial heterogeneity (I² = 63%; Chi² = 13.65, p = 0.02). These results are consistent with the main analyses, suggesting no impact of PNF on symptom severity for this follow up period.

There were insufficient studies available to conduct further sensitivity analyses.

### Forest plots for mixed PNF studies

E1: PNF+ vs passive control: 0-3 month follow up

E1.1 Frequency of behavior


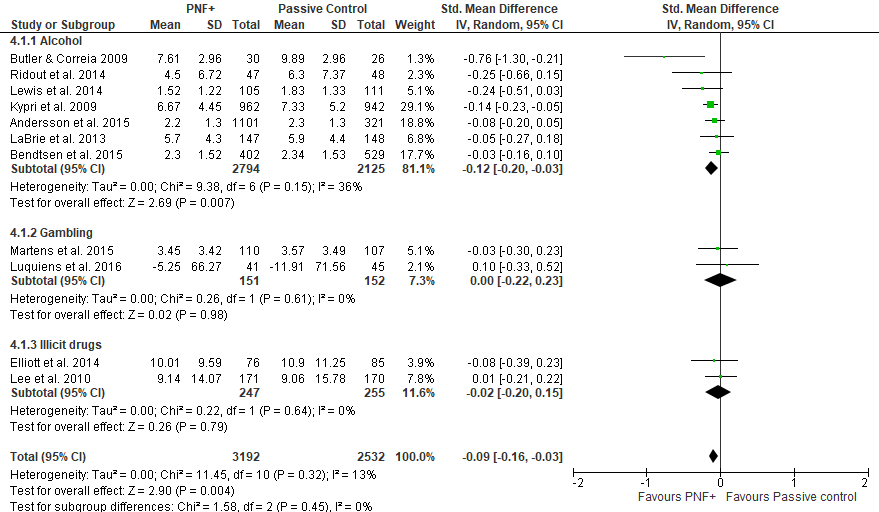


E1: PNF+ vs passive control: 0-3 month follow up

E1.2 Symptom severity


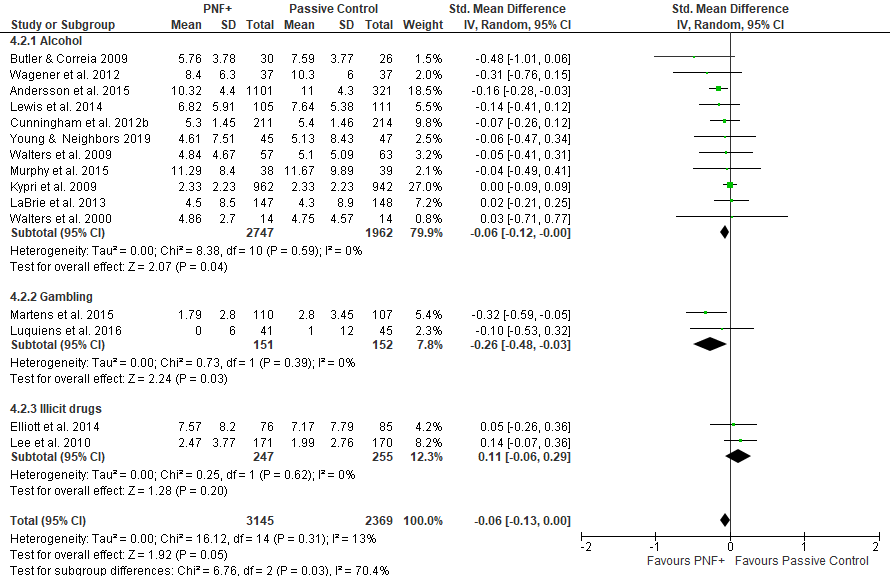


E2: PNF+ vs passive control: 4-11 month follow up

E2.1 Frequency of behavior


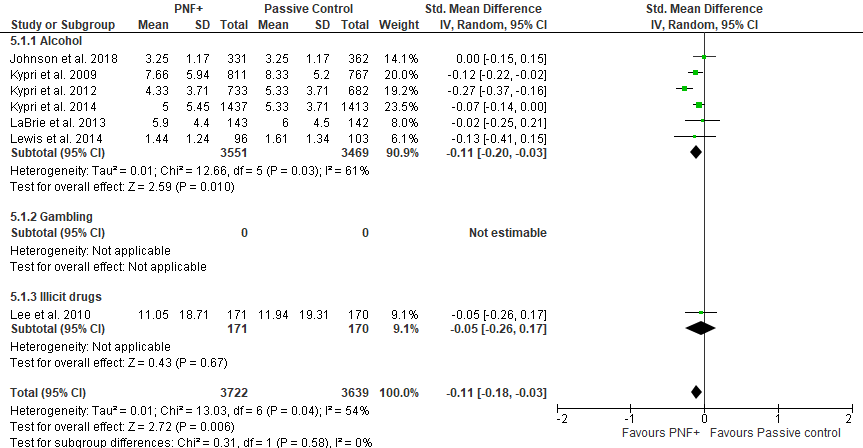


E2: PNF+ vs passive control: 4-11 month follow up

E2.2 Symptom severity


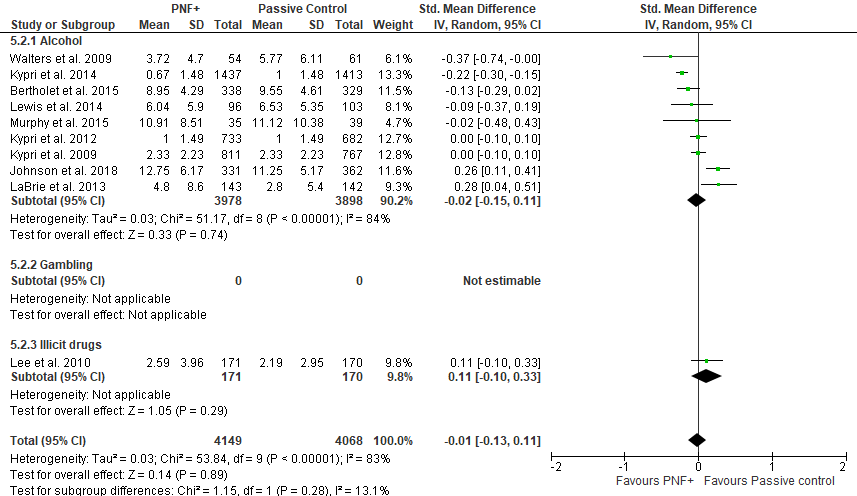


E3: PNF+ vs passive control: 12-23 month follow up

E3.1 Frequency of behavior


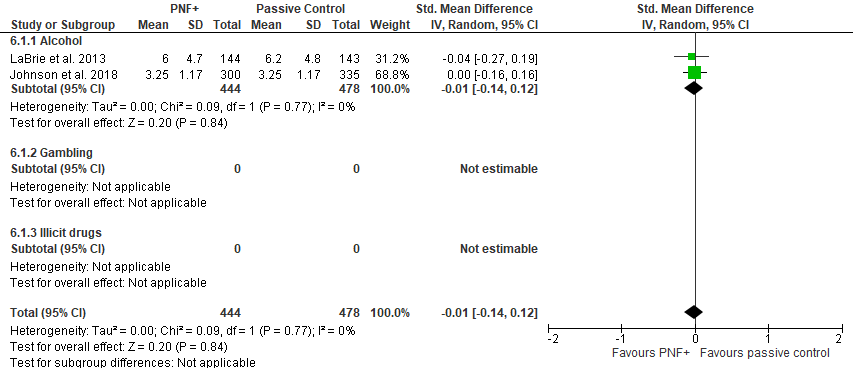


E3: PNF+ vs passive control: 12-23 month follow up

E3.2 Symptom severity


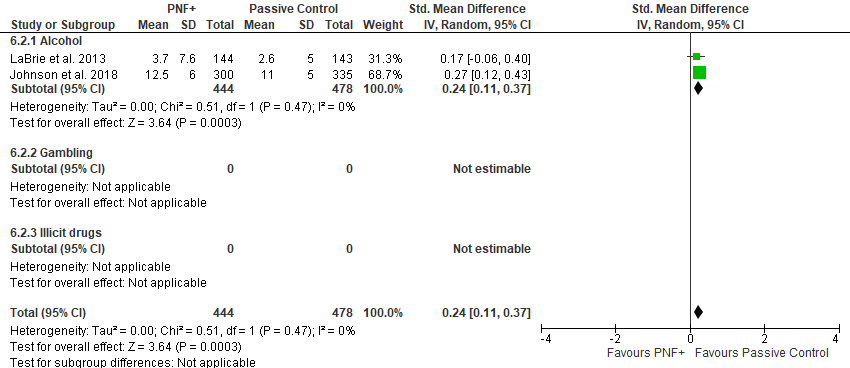

Supplement: S5 Appendix — (DOCX) [file pone.0248262.s006.docx]
