# Supplementary material for: The efficacy of Personalized Normative Feedback interventions across addictions: A systematic review and meta-analysis
Source: PLoS One. 2021 Apr 1;16(4):e0248262. doi: 10.1371/journal.pone.0248262 (PMC8016245; doi:10.1371/journal.pone.0248262)
Supplement: Attachment — Submitted filename: PLOS one response to reviewers.docx [file pone.0248262.s007.docx]

Reviewer #1: Saxton, Rodda and colleagues investigated through a systematic review followed by meta-analysis, the efficacy of Personalized Normative Feedback (PNF) alone, and with additional interventions (mixed PNF interventions) for hazardous alcohol use, problem gambling, illicit drugs use and smoking. Thirty four studies were included on systematic review which thirty had suitable data for meta-analysis. They found that PNF alone and mixed PNF interventions were effective in reduction short-term alcohol consumption and symptom severity. In gambling problem, mixed PNF interventions reduced short-term symptom severity. Data from illicit drugs and smoking was not enough to assess the efficacy of PNF in these cases.
The study was well designed and achieved the goals proposed by the authors, but there are some minor issues that need to be addressed to improve the manuscript.

*Thank you to the reviewer for their positive response to the manuscript.*

I missed a brief historical contextualization of PNF. You may include who proposed and validated the PNF for the first time, mainly for behavior addictions.

*- Regarding the historical context we have now included a paragraph on the origins of PNF for addictive behaviours.*

Review rationale

DSM-V was published in 2013 considering addiction problems as substance use and gambling. However, on page 481 there is an introduction text wherein they say “Other excessive behavioral patterns, such as Internet gaming, have also been described, but the research on these and other behavioral syndromes is less clear. Thus, groups of repetitive behaviors, which some term behavioral addictions, with such subcategories as "sex addiction," "exercise addiction," or "shopping addiction," are not included because at this time there is insufficient peer-reviewed evidence to establish the diagnostic criteria and course descriptions needed to identify these behaviors as mental disorders”.
Regarding there are several studies showing shared neurobiological mechanisms among drug use, gambling and other behavioral addictions, why did you not include this terms on systematic search? If was a personal choice I think you could include an explanation about that. Why not include other behavioral addictions?

*- We agree with the reviewer that other addictions would be very interesting. PNF requires re-presentation of population norms and given the relative newness of other behavioural addictions we did not expect to find any studies. This meant we decided to include only those disorders currently classified in the DSM -5. We acknowledge, however, that future iterations of this review should include the newer behavioural addictions.*

Search strategy: Better explanation why the year interval was from 2000 to 2019.

*- By adding the brief history as suggested by the reviewer we believe we have now addressed the timeframe. We have added this rationale to the search strategy.*

Clarify how you conducted the systematic search. How did you combine the keywords? Why did you use different terms in different databases? You could improve the design of appendix 1.

*We have edited the search strategy in text (lines 188-190) for clarity. We believe this explains the combination of keywords. Re the use of different terms in different databases, we understand that mesh terms differ across different databases. We have, where possible, maintained the same keywords for each search*.

Reviewer #2: By the abstract, the study aim seems interisting but tricky: to examine the efficacy of Personalized Normative Feedback (PNF) interventions upon alcohol, gambling, illicit drugs and smoking disorders and, to be widely used for all the this four disorders at the same time, once it is a low-cost intervention strategy. However the authors failed to produce an effective statistical analysis and satisfactory discussion and conclusion that could explain their results. Also, is important to be registered that the protocol and/or parts of this review was sent (but not published, according to the website) in PROSPERO database and is under review since 2018.

*- The reviewer notes that the review was registered in 2018. We believe this is not an unusual amount of time to undertake a complex review. Moreover, the search was updated prior to submission with PLOS One. With regards to the comment that we are suggesting PNF is used for all four disorders at the same time – this was not suggested in the article. Rather, we are arguing that this type of brief intervention could be used for all disorders classified in the DSM-5 as addictions. With reference to the results, we address this below where the reviewer has more fully expressed their concerns.*

The text is full of gramatical problems, not appropriate words (e.g. usage of the word "hazardous" instead of alcohol use disorder, usage of the word "setting" instead of social insertion, usage of the sentence "illicit drug practices" instead of illicit drug disorders) and confused in many parts, making reading really difficult. Also, along the text the authors use the word "addiction" instead of "disorder" (or use in few moments) that is what PNFs are used to identify: disorders related to substance seeking and consumption.

*-We have reviewed the manuscript to ensure wording is consistent throughout. In particular, we have removed the words ‘practices’, addiction (where less appropriate). However, our eligibility criteria included study samples that consisted of individuals with some level of problematic use of alcohol, gambling, drugs, or tobacco use at baseline. The term “hazardous use” has therefore been retained as this is a term used in the PNF literature to refer to problematic levels of alcohol consumption that do not necessarily constitute alcohol use disorder (see reference list for example).*

The first 2 serious concerns about the paper are conceptual errors: the first is that the authors affirm in "Review Rationale" that they aimed understand if PNFs could be used as a candidate intervention for treatment of all the four disorders. However, PNFs is widely known for being a brief intervention tool that are not expected to be used as full treatment but as a personal screening tool to identify individuals that need further interventions (psychological, psychiatric) related to seeking and consumption disorders (the authors cite that in page 53 using another authors as reference, however is not possible to identify if it is also their opinion because they write shortly thereafter that PNFs could be used as "standalone intervention");

*- We have removed reference to ‘treatment’ and retained intervention so as to differentiate PNF as a brief intervention from more intensive treatment approaches. We offer different ways PNF could be used and as stated by the reviewer, this could be as a means of identifying individuals that may be interested in face-to-face treatment. However, as indicated in multiple studies and our review, PNF can also have an impact of frequency of consumption and severity of symptoms as a standalone intervention. The wider literature and evidence in our review does not support the reviewers assertion that PNF should primarily be used for screening and referral only. We strongly disagree that this is a conceptual error and we have presented multiple perspectives on how PNF might be applied.*

The second conceptual error is that the authors also affirm in "Review Rationale" that alcohol, gambling, illicit drugs and smoking disorders share "commom mechanisms" and used that sentence as a justification for the use of a common PNF for all this disorders. We have a problematic things in here: What the authors call as common mechanisms? The authors they used as a reference say that behavioral and substance addiction share etiological, phenomenological and clinical mechanims and the only one related to a neurobiological perspective is dopamine dysregulation. Further, the authors used as reference also affirm that there are neurobiological differences between behavioral and substance addiction (and give some examples). This (and all the literature related to drug disorders and addiction) leads us to think that an PNF (pure or mixed) would be tricky and not be effective to be used for all the four disorders discussed in this review, because there are many neurobiological, social, age, culture, sexual and several other aspects to be considered related to those disorders and addictions.

*- We are not arguing that there is a common tool for all disorders, rather that PNF as an intervention type could be used for all addictive disorders. The actual intervention items and norms should be based on the specific area of interest. Moreover, the aim of the study is to identify whether indeed PNF can be employed successfully for each of these addictive behaviours and our analyses allow us to draw conclusions on the possible differential efficacy of PNF on these behaviours.*

One of the aim of the authors, and that they say makes this review different from the protocol sent to PROSPERO, is that they used smoking disorders as one of the PNFs used for this disorder. However, there is any reference that was found and used in this review.

*- The reviewer is correct, we are now including tobacco use in our group of addictive behaviours. To our surprise, we did not locate any studies that used pure or supported PNF. Given it was in our search, it is not appropriate to remove it for publication. Moreover, the gap identified in this field of research is an important outcome of this study.*

Also, it was not clear why the authors used controlled prescription substances (such as sedatives, benzodiazepines and ketamine) and "designed drugs" in their search terms - there are no mentions about why they considered it and why they searched but did not mention that along the text.

*- The review was inclusive of all illicit and prescription drugs and therefore these substances were included.*

One of the "Study Eligibility Criteria" was that the studies had at least one outcome (frequency and/or sympton severity). However, frequency without quantity is subjective and it was not explained why they did not use both consumtption patterns.

*- When examining multiple substance and behavioural addictions, it is not possible to consistently identify an index of quantity. This is because quantity in some addictive behaviours (e.g., gambling – where expenditure could be the quantity index) means quite a different thing to other addictive behaviours (e.g., alcohol – where number of standard drinks may be the quantity index). We therefore selected frequency and symptom severity as the key outcomes. We have included this statement in the methods- study eligibility section.*

Further, they justified that they excluded outcomes measuring quantity-type and binge-related outcomes because they could measure "different construct" - it is not clear what that means once most studies use AUDIT and other similar tools to measure the amount of drinks, for example.

*- Excluded studies that examined binge-related outcomes may measure frequency but this is often in terms of frequency of binges rather than frequency of use. Moreover, data on binges and quantity (as above) do not consistently apply over all substance use and behavioural addictions*

And in the discussion session they write about the difficulty to find other reviews that use frequency outcomes as a criteria (making it clear that studies usually prefer using quantity intead of frequency).

*- The statement regarding limited reviews examining frequency was specific to gambling (line 867). The lack of other reviews which included frequency was due to gambling focusing more on expenditure and severity. Expenditure is not a viable comparison in our study where we included multiple substance and other additions. We also noted this requirement as a limitation on line 973.*

Also, they used sympton severity as another outcome without explaining why and the criteria used to chose it as a study criteria.

*- As noted above we have now included a sentence in the methods section stating reasons for the use of frequency over quantity. That is, we selected frequency and severity as they are indices that can consistently be applied across all of the included substance and behavioural addictions.*

The authors also used 3 studies that could be argued to be selected as an exclusion criteria, as they wrote in page 9: one study with war veterans, one study in an army recruitment center and, another in outpatients waiting room; there were no explanations why there were used.

*- The exclusion criteria as stated on page 260 was “PNF intervention targeted people with specific physical or psychological comorbidities (e.g., war veterans with post-traumatic stress disorder), which are less generalizable to other populations”. This is not exclusive of studies that involved veterans rather those with physical or psychological comorbidities.*

Also, the authors used as an exclusion criteria people with specific physical or psychological comorbidities, however they did not explained if the studies they used all the participants had or had not comorbidities (one aspect that is super common in individuals with drug-related disorders).

*- As stated above, they were excluded where the intervention targeted this group. We did not exclude intervention who reported psychical or psychological comorbidities in the participant characteristics because we agree that these comorbidities are highly relevant to the examination of these disorders.*

There are no diversity among the populations, nationality and ages in the studies used as reference. In "Pure PNF" tables is used only studies from the USA and in female young University population. And in "Mixed PNF" tables also most studies are from USA and in young University population. In most those studies there are only one outcome (frequency or severity), in the majority there are no effective results and, basically all the studies are alcohol-related disorders papers (among the 37 papers used as references, only 3 are about gambling and other 3 with marijuana - and not illicit drugs as the authors used to refer it). However, the authors insisted along the results, discussion and conclusion that "...it is also the first review to provide evidence about the efficacy of PNF across multiple addictive behaviours and settings..." (page 51).

*- As noted by the reviewer, we were answering a set of a priori questions that were published on Prospero. The evidence that we provide in this review does not just relate to the efficacy of PNF, but also allows us to note an absence of empirical evidence, across the four substance and addictive disorders. In emerging fields, such as gambling, it is important for reviews to identify gaps in the evidence base, thus calling for the need for researchers to redress these gaps.*

The same authors used the sentence "...but there was a lack of studies focused on illicit drugs, few on gambling and, none on smoking which limited our statistical power. Without additional studies in these areas, we cannot draw firm conclusions about the utility of PNF across addictive behaviors." (page 44).

*As above, we were very careful not to speak beyond the regarding the efficacy of PNF for these conditions. As stated by the reviewer, the research identified a significant gap in this area and we have highlighted this gap for the consideration of future research.*

Also, in page 54 the authors are not conclusive about their opinion if PNF could be or could be not be used, as they propose, "a standalone intervention". Considering those conflicting sentences and the statistical methods used (explained below), it is clear that the authors are not sure about their results and conclusions and, is also clear that the complexity of the drugs abuse disorders and all the other factors involved is not under consideration in this review.

*- We have stated “PNF could be a useful kick start to more intensive intervention as necessary, or as a standalone intervention for people at the lower end of the risk continuum to get people motivated and engaged to change their practices.” We have now added a statement in the discussion that future research is required to investigate this possibility.*

About the statistical analyses, in the beggining of the text the authors write that they converted medians to "means" (average) but it is not clear why.

*- Where studies only provided medians, we converted these medians to means so as to allow the meta-analysis to be conducted (see page 13 of the manuscript). As indicated in the manuscript, this is not yet standard practice for systematic reviews, but it is an acceptable approach based on the Cochrane Handbook for Systematic Reviews of Interventions (Higgins & Deeks, 2011) (see Higgins JPT, Deeks JJ (editors). Chapter 7: Selecting studies and collecting data. In: Higgins JPT, Green S (editors), Cochrane Handbook for Systematic Reviews of Interventions Version 5.1.0 (updated March 2011). The Cochrane Collaboration, 2011. Available from* [*www.handbook.cochrane.org*](http://www.handbook.cochrane.org)*.*

Also, in page 18 (item h) the authors write "Means and SDs were not extractable from the paper due to poor resolution of graphs", this leaded me to ask: How did they collect all the other results? Did they collect all the data used in this review from graphics? If so, this compromises all the review results, discussion and conclusion.

*-Data was sourced and extracted from text and tables and graphical illustrations in rare cases (this is noted on page 13). In Table 1, we noted for this specific study (reference number 68), there was no outcome data reported in the text or table and the graph could not be interpreted.*

Also, in almost all the statistical analyses they removed studies (most of them the ones with no effect or no results about the frequency and/or severity) and this also compromises this review. The question in here is: Why not using the data the are available in the papers used as reference?.

*- A meta-analysis requires data in order to be conducted. As stated by the reviewer, these excluded papers did not report frequency or severity outcomes (often descriptive papers only).*

Finally, in page 51 the authors write "Studies of other illicit drugs are notably absent, and researchers continue to be challenged by recruitment and other practical obstacles to adressing behaviors that participants know are agains the law". Behaviors related to drug use, abuse and addiction are not against the law, drug traffic is against the law. Drug abuse disorders and drug addiction is a serious world health problem and individuals that suffer with it must be treated with empathy and kindness once it is a disease just like any other and treatment is necessary. Most of them are marginalized populations with no access to doctors and multidisciplinary strategies to treat their illness. Most of them do not know that their condition is a disease because the sociaty still stigmatizes their illness as a character flaw. Participants maybe do not easily share their disorders related to drugs use/abuse because they are treated as outlaw persons and not as what they really are: people who need adequate treatment and attention.

*- We do not disagree with the reviewer about the conceptualisation of these behaviours as a public health issue. We were simply highlighting a possible methodological reason for a lack of studies in this area. However, to acknowledge the reviewer’s comment, we have removed the statement related to legal issues associated with illicit drug use.*
