# Supplementary material for: The efficacy of Personalized Normative Feedback interventions across addictions: A systematic review and meta-analysis
Source: PLoS One. 2021 Apr 1;16(4):e0248262. doi: 10.1371/journal.pone.0248262 (PMC8016245; doi:10.1371/journal.pone.0248262)
Supplement: Attachment — Submitted filename: plos one response to review Dec submitted.docx [file pone.0248262.s008.docx]

6. Review Comments to the Author

Reviewer #1: Saxton, Rodda and colleagues re-submitted the paper about systematic review followed by meta-analysis of the efficacy of Personalized Normative Feedback (PNF) alone, and with additional interventions (mixed PNF interventions) for addictive behaviors. Authors provided good answers for concerns highlighted by the reviewers, but there isa minor issue that needs to be addressed to improve the manuscript.

*Thank you for the positive feedback*

In introduction, the authors affirm, based on literature, that all addictive behaviors share a common mechanism. In Results they found differences in effects of pure and mixed PNF interventions for alcohol and gambling, but no influence for illicit drugs and tobacco use. I think authors could suggest why these differences appeared between each addictive behavior.

*We have now added a section into the implications section on the role of importance and readiness across different addictive behaviours that may, in part, explain these findings.*

Reviewer #2: Despite the reviewers questions and sugestions made, the authors realized just few changes along the text and did not responded in a directed manner and/or were vague in their responses. I consider that the material that was presented and, is now once again presented without big changes, is not suitable for publication in PlosOne at this moment. Below I list point by point the reason of my final decision reviewing this paper.

*Thank you Reviewer 2 for the time they have spent on our manuscript. We have carefully reviewed their first and second round of comments and strongly refute the claim that without big changes the manuscript is not suitable for publication. The PLOS One guidelines state peer review is used “to determine whether a paper is technically rigorous and meets the scientific and ethical standard for inclusion in the published scientific record.” We believe that we have met and exceeded these standards as indicated by three of the four reviewers. We have ensured the work is aligned with established standards relevant to systematic reviews including PRISMA and Cochrane. Our study was also registered with PROSPERO prior to its undertaking and we have undertaken our analysis consistent with the registered protocol.*

#1 - The first reviewer asked for a historical contextualization of PNF (including who proposed and validated). The authors answered they included a paragraph in the text, however this paragraph is consuding and does not clearly explain what was sugested and the importance of PNF (why it was first proposed, for example).

*As indicated by Reviewer 1, the historical contextualisation of PNF that we provided was satisfactory. As stated in the text, PNF was first developed in the US in response to college student drinking.*

#2 - The first reviewer asked for the authors to clarify how the systematic search was conducted and also asked for improvements in Appendix 1. The authors answered they edited the search strategy, however nothing was noticed in the "new" text and no improvements were made about the Apendix 1 design.

*Apologies for the confusion. These changes were made in the first revision in response to Reviewer 1 but the relevant changes were not highlighted in the manuscript.*

#3 - I mentioned that aparently the authors suggested that PNF was proposed in this review as a tool intervention for all the fours disorders at the same time. The authors answered they did not suggest that along the text. Considering that, the authors should better look at the lines 107-110 and 125-131 and rewrite the sentences to better clarify what is proposed by them.

*Thank you we have clarified that the intention is to look at the use of PNF for each of these different addictive disorders – not as one treatment.*

#4 - I suggested a gramatical problem along the text and suggested few words to be changed for more appropiate words. The authors answered they reviewed the text and removed the word "practices". Indeed the authors changed the word "practices" for more appropiate words, however the text does not seem that passed throug a gramatical review. The text is still confusing along the review.

*The text has been reviewed and any grammatical errors have been amended.*

#5 - I commented about what makes this review presented to PlosOne different from the one submitted to PROSPERO (the use of smoking studies) and the absence of references about smoking studies in this review. The authors answered they included tobacco use in their groups of addictive behaviours and that they did not find any new studies, just like in the smoking related studies they did before. However, the authors did not present a new Appendix and it seems they just switched the work "smoking" for "tobbaco" along the text with no justification.

*The reviewer is correct that the Appendix reflects the search which was conducted as stated. As noted in the search terms both tobacco and smoking were used. Changes in the text were in response to the reviewers request for consistency in terms*.

#6 - I highlighted that the authors were not clear in the text about the use of controlled prescription substances and "designed drugs" in their search. This question remains unsolved, the authors did not anwered the question and did not include anything in the text that could clearfy this information.

*The Study Eligibility Criteria section of the manuscript has been edited to clearly indicate that the review was inclusive of all illicit and prescription drugs. The search terms are not able to be changed now.*

#7 - I asked why the authors chose frequency and/or severity instead of quantity. The authors answered explained their option for using frequency and severity and included it in the text. However, the authors affirmed those are powerfull tools without using any reference that could confirm that the use of frequencyand/or severity is more suitable than quantity.

*To clarify, in the manuscript we did not state that these tools were more powerful, rather we stated that there are problems when making comparisons between alcohol, drugs, gambling and smoking in relation to quantity. In behavioural addictions, such as gambling, quantity is not easily comparable to other addictions such as quantity of alcohol consumption, hence the decision to only explore frequency and severity.* *This is consistent with other reviews of substance and behavioural addictions in which symptom severity and frequency have been the primary outcomes of interest (see example below). These have also been added in the manuscript.*

[*https://www.frontiersin.org/articles/10.3389/fpsyt.2018.00095/full?report=reader#h3*](https://www.frontiersin.org/articles/10.3389/fpsyt.2018.00095/full?report=reader#h3)

#8 - Along the responses to the questions made, the authors demonstrated that the aim of the review would be demonstrating "the importance of reviews to idenfity gaps in the evidence base, thus calling for the need for researchers to redress these gaps". This is what the authors should have proposed once their results are basically related to it. However, the authors affirm along the text that this review presented confirm the importance of PNF despite the gaps found, and sometimes affirming its efficacy taking in consideration just one study (for gambling). I still sugest to the authors to review their aims for this review, I believe those aims clould be changed to a better result and better discussion.

*The aims are as stated in the Prospero registration and therefore cannot be changed. We have re-read the results and discussion and believe that we have interpreted the results of the meta-analysis correctly and not overstated the conclusions.*

#9 - I stated that the the authors opted to convert the medians of the studies used into medians, and this choose was not clear. The authors answered that they did it to allow the meta-analysis. Moreover, the authors answered that this is not the standard practice for systematic reviews but acceptable according to their reference, without explain why they chose this option instead of the standard practice for systematic reviews.

*As stated in our previous response, the Cochrane Handbook for Systematic Reviews states that this is an acceptable approach. To ensure this process was rigorous we also reported additional sensitivity analyses to assess whether papers for which we converted medians to means affected our findings. Note that the results of these sensitivity analyses were mostly consistent with the main findings, suggesting that this approach did not have a major impact on the results.*

#10 - I noticed that the authors used data derivative from graphics and this was one of the points I highlighted, hoping it was just a misunderstanding. However, the authors affirmed my suspicions answering that in fact they extracted some data from graphical illustrations in rare cases. In my point of view, this is a wrong way to collect data and may compromise the final results.

*This was done for one study where no data was provided in the text. As stated in the Cochrane guidelines, extracting data with a ruler from a figure is an acceptable approach. https://training.cochrane.org/handbook/current/chapter-05*

#11 - Finally I had highlighted a sentence that was completely wrong and conceptually misunderstood - "Studies of other illicit drugs are notably absent, and researchers continue to be challenged by recruitment and other practical obstacles to adressing behaviours that participants know are against the law". I spent time explained in few lines how wrong this sentence is, but it looks like that the authors do not realize how stigmatized and wrong this sentence is once they changed this sentence for "Studies of other illicit drugs are notably absent, and researchers continue to be challenged by recruitment and other practical obstacles to addressing illicit behaviors". I will not extend myself explaining it again, it was well explained the other time, with no understanding by the authors. My concern is that this sentence was not a wrong way to express the gap in literature relating PNF and illicit drugs, but that this is the authors opinion about people with drug-related problems.

*We have changed this to refer to people using other illicit drugs so as to avoid any implied stigma, and have removed reference to this being an illicit behaviour.*

Reviewer #3: The systematic review and meta-analysis by Saxton, Rodda and colleagues assessed the efficacy of 'pure' personalized normative feedback (PNF) interventions and in combination with additional self-directed interventions (‘mixed’ PNF) on frequency and symptom severity of hazardous use of alcohol, tobacco, illicit drugs and problem gambling. Authors reported significant effects of 'pure' and ‘mixed’ PNF in reducing short-term frequency and symptom severity associated with alcohol use. In addition, they observed that ‘mixed’ PNF reduced gambling symptom severity in the short term. No effect of the intervention was found on outcomes associated with illicit drugs use. No tobacco study met the inclusion criteria. Authors discussed the importance of conducting future studies to examine whether PNF could be a useful kick start tool to more intensive intervention, or a standalone intervention in some cases. Authors also discussed the need for future cost-effectiveness research to evaluate whether application of PNF in a more comprehensive manner is worthwhile.
I consider that the manuscript is well written, the aims are well defined, methodology is clear and conclusions are supported by data. Authors appropriately discussed the strengths and limitations of the study.
I believe that the previous review brought improvements to the manuscript.

*Thank you for your feedback on our manuscript*

I would like to point out some minor issues and suggestions:

1. Please, include in the abstract what the acronym “RCT” means (= randomized controlled trial).

*The change has been made to the abstract to spell out RCT.*

2. In the abstract, authors conclude, “Our findings highlight the efficacy of PNF to address alcohol frequency and symptom severity”, but what about gambling, since they affirmed “PNF with additional interventions reduced short-term gambling symptom severity”. I understand that authors did not draw a firm conclusion about the effect of PNF on gambling symptom severity due to the reduced number of gambling studies included in the analyses, but I think that this could be explained in the abstract.

*We have now addressed this in the abstract by highlighting this issue with reduced number of studies.*

3. Suggestions: on page 3 (introduction), authors could maybe introduce drugs of abuse topics first (alcohol, tobacco, illicit drugs), and then introduce gambling-related topic.
In addition, on page 10, I think authors could place the sentence “We selected frequency and severity as they are indices that can consistently be applied across all of the included substance and behavioral addictions” after mentioning both evaluated outcomes, frequency and symptom severity (at the end of the paragraph).

*We have altered the order of the topics at the start of the introduction as well as the order in the first paragraph. As suggested, we have also relocated the sentence on page 10 to the end of the paragraph.*

4. On page 10, authors could include a brief explanation about why exactly they decided to exclude quantity measures, BAC, attitudinal change and dollars spent on gambling.

*We have added the following explanation on page 10: ‘In relation to symptom severity we excluded quantity measures, blood alcohol content (BAC) and dollars spent on gambling because they are addiction-specific and not applicable across the range of substance use and behavioural addictions our review included. We also excluded measures of attitudinal change because our aim was to assess the effect of PNF on behavior change. We focused on frequency and severity outcomes as they are indices that can consistently be applied across all of the included substance and behavioral addictions.’*

5. I think that table 5 (page 38) may have formatting issues.

*Thank you the column had narrowed and separated the minus signs from the text. We have corrected that now.*

6. In the “Implications for clinical practice” section (discussion), authors discuss about whether PNF effects in reducing alcohol frequency and symptom severity are meaningful or not, due to the small effect sizes observed. About this, authors brought considerations that were addressed by other reviews. However, it was not clear to me the authors’ opinion on this.

*We have reworded the next sentence so as to present evidence to support intervention research that produces moderate effect sizes.*

7. In the “Follow up period” section (discussion, page 52), it would be nice to discuss more about the long-term effect of mixed PNF on alcohol symptom severity favoring the control group. Did authors hypothesize why this happens?

*Reviewer 3 raises an interesting question. The two long-term alcohol studies were LaBrie et,al 2013 and Johnson et al 2018, and both also report medium-term findings. Forest plots show their findings are already favouring the control group at this earlier stage, in contrast to the other seven studies in the medium-term group. This leads us to conclude their results at 12-23 months were a continuation of the effects seen at 4-11 months. Though there are a number of possible reasons why these two studies demonstrate more favourable outcomes in the control group (e.g., despite randomisation, pre-existing differences between the intervention and control groups affected the results), we feel the finding highlights the caution needed in drawing firm conclusions when relying upon only two studies. We have checked the manuscript to ensure this caution is highlighted for results based on two studies, and have added text to reflect this in the ‘Comparison to wider literature’ section of the discussion.*

Reviewer #4: Saxton and colleagues report a systematic review and meta-analysis of the efficacy of Personalized Normative Feedback (PNF) interventions across addictions in meticulous detail based on about 30 studies. This revised manuscript represents a carefully conducted study on an interesting, albeit rather weak intervention to address a variety of addictions. The authors were
responsive to the comments from previous reviewers and made necessary changes, while avoiding recommendations that were inconsistent with their registration of the study in Prospero.

*Thank you for your positive comments on our manuscript*

The write up of this study was very complete (~65 pages plus supplemental material), but tedious to read. It seemed that many paragraphs were essentially redundant with the substitution of another analysis or sub-analysis with slightly different statistical results. In contrast to Tables 3-6, I found that the forest plot figures in the Supplemental Material (Appendices D and E) really told the whole story. They present the meta-analytic results clearly and much more succinctly than the pages of narrative that were highly redundant with the Tables. Although the figures would need to be redone to save space, the graphic depiction of results is rather compelling, negating the need for the repetitive statistical results format presented.

*Thank you to the reviewer on their suggestion to replace the tables with forest plot figures. We agree this is a much improved representation of the findings and have now removed the tables and replaced them with forest plots. To save space, these forest plots were re-done to include only the results of the main analyses. All subgroup analyses have now been reported in text.*

The discussion was a rather lengthy recap of results, some of which did not need to be repeated. After focusing on effect sizes, I was anxious to read the authors’ interpretation of the extent to which PNF produced clinically- or societally-meaningful improvements in health and wellbeing. I felt the discussion pertained more to the limitations of the available research, or lack thereof, than the intervention itself. What I took away was that perhaps this may be cost-effective because the small and short-term effects (never clearly defined) are offset by the low cost of the intervention.

*Thank you to the reviewer for highlighting the repetition. We have removed the ‘strengths’ section from the limitations and focused this section solely on limitations of the current paper. We also removed 3 paragraphs (400 words) on limitations of the literature, which are discussed elsewhere.*

In summary, I believe this review is worthy of publication. This is a carefully done review and meta-analysis and it more than adequately summarizes what is known about the efficacy of Personalized Normative Feedback interventions. However, I am not sure that it warrants such a lengthy treatise.

*Thank you to the reviewer for their comments.*
